# High-quality draft genome sequence of the tropical lichen-forming fungus *Lecanora helva* (Lecanoraceae, Ascomycota)

**DOI:** 10.1128/mra.00610-25

**Published:** 2025-10-13

**Authors:** Yukun Sun, Meredith M. Doellman, Alejandrina Barcenas-Peña, Vasun Poengsungnoen, Sabine Huhndorf, H. Thorsten Lumbsch, Felix Grewe

**Affiliations:** 1 Grainger Bioinformatics Center, Collections, Conservation and Research Division, The Field Museum 635107 , Chicago, Illinois, USA; 2 Lichen Research Unit, Department of Biology, Faculty of Science, Ramkhamhaeng University, Bangkapi, Bangkok, Thailand; University of California Riverside, Riverside, California, USA

**Keywords:** Lecanora helva, lichenized fungi, whole genome assembly, telomere-to-telomere assembly

## Abstract

We present a high-quality genome assembly of the lichenized fungus *Lecanora helva* collected in a tropical mangrove in Thailand. Using PacBio HiFi sequencing and a novel multi-assembler approach, we generated a contiguous, nearly complete (30.07 Mb, 98.1% BUSCO completeness) genome sequence, enabling future studies on mangrove adaptation and climate resilience.

## ANNOUNCEMENT

*Lecanora* is one of the largest genera of crustose lichens, with a worldwide distribution (1, 2). We sequenced the genome of the fungal component (mycobiont) of *Lecanora helva* Stizenb., a tropical lichen species potentially sensitive to climate change. The specimen was collected on 07 August 2021 from the bark of *Avicennia alba* Blume in a mangrove forest in Tambon Lat Khwang, Amphoe Ban Pho, Chachoengsao Province, Thailand (13^o^36’41.9”N, 101^o^02’21.4”E), and deposited in the Lichen Herbarium of Ramkhamhaeng University (accession: RAMK 036147; collection number: VPMG_37; herbarium database: http://www.lichen.ru.ac.th/images/data/STT/STT46_Lichen%20herbarium%20database%20and%20management.pdf). An axenic fungal culture was produced from a single ascospore and grown on malt-yeast extract agar under laboratory conditions (3).

High-molecular-weight DNA was extracted from the axenic fungal culture using a modified protocol in Wilken *et al*. (4) and sent to the UWBC DNA Sequencing Facility. DNA quality and quantity were measured using a NanoDrop One (ThermoFisher Scientific, Waltham, MA, USA), a Qubit dsDNA High Sensitivity kit, and a FemtoPulse System (Agilent, Santa Clara, CA, USA). A PacBio HiFi library was prepared using protocol PN 102-166-600 APR2022 (Pacific Biosciences, Menlo Park, CA, USA), with modifications including Covaris gTUBE shearing (Covaris, LLC, Woburn, MA, USA) without further size selection. Library quality was assessed using the FemtoPulse, and the library was quantified using the Qubit dsDNA High Sensitivity kit. Sequencing was performed on a Sequel II using the Sequel Polymerase Binding Kit 2.2. A total of 620,494 raw reads were generated (N_50_ =8148); adapter contamination (“AAGCAGTGGTATCAACGCAGAGTACT”) was removed with lima v.2.7.1, and duplicates were marked with pbmarkdup v.1.0.3 (5).

The assembly was constructed with a novel consensus-based strategy combining outputs from multiple assemblers. PacBio HiFi reads were assembled into unphased contigs using six tools: Canu v.2.2 (6), NextDenovo v.2.5.2 (7), IPA (8), Flye v.2.9.5-b1801 (9), Peregrine-2021 v.0.4.13 (10), and RAFT-hifiasm v.0.19.9-r616 (11–14). The Peregrine-2021 output was selected as the primary assembly due to its lowest contig number, highest N_50_, and greater number of identified telomeric ends, indicating superior genome contiguity. To obtain additional telomere-to-telomere contigs, contigs with telomeric sequences at one terminus from alternative assemblies were compared and merged with QuickMerge v.0.3 (15). Resulting contigs were manually curated; Redundans v2.0.1 (16) was used for redundancy reduction. Genome contiguity and completeness were assessed using QUAST v.5.3.0 and BUSCO v.5.7.0 with the ascomycota_odb10 data set (17, 18). Repeats were identified and masked before annotation using RepeatModeler 2.0.1 (19), followed by RepeatMasker 4.1.2-p1 to mask repetitive elements (20). Annotation was performed using Funannotate v.1.8.17 and InterProScan v.5.72-103.0 (21, 22). Default parameters were used except where otherwise noted.

**Fig 1:**
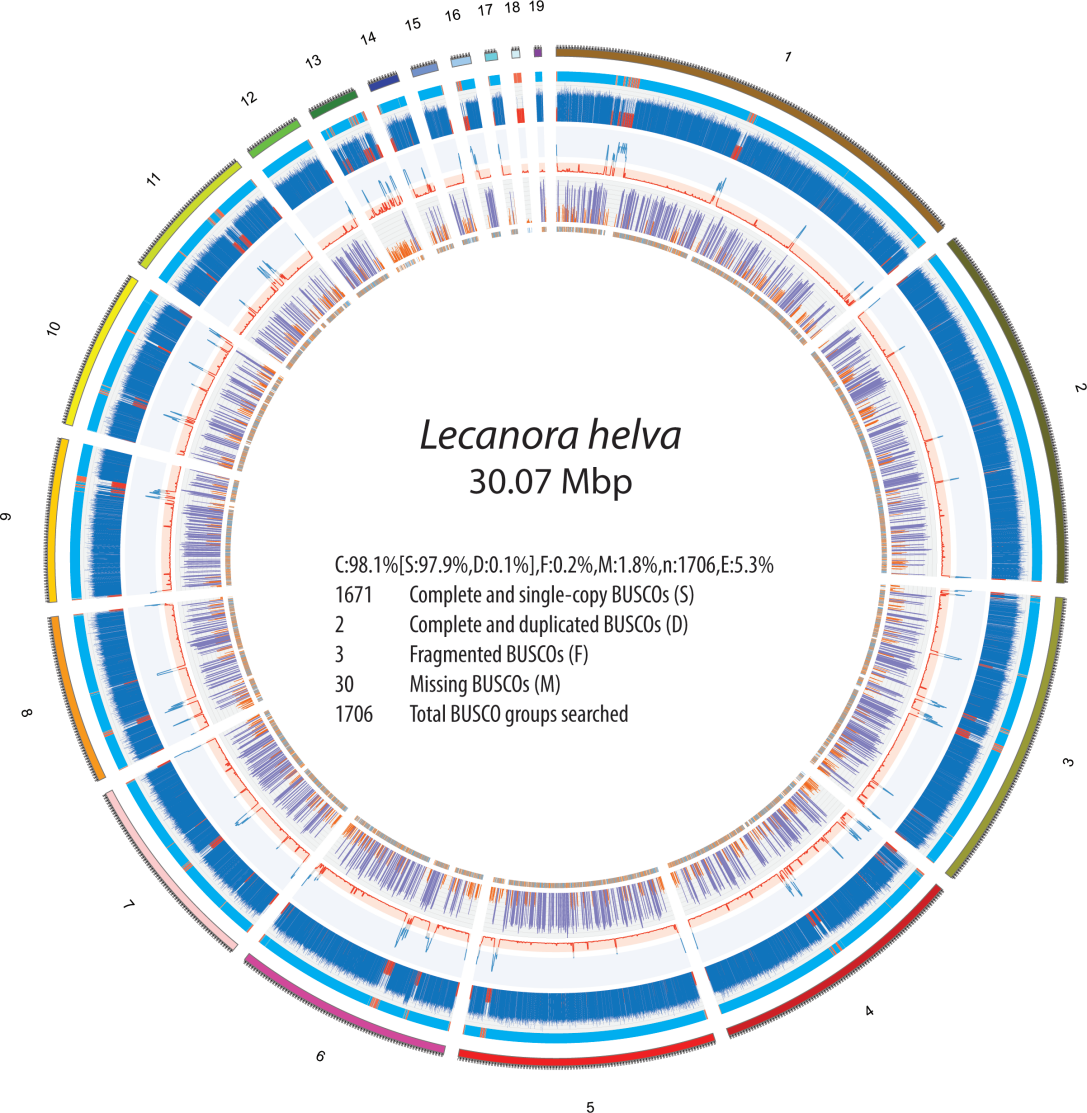
Circular representation of *L. helva* genomic features. The plot comprises concentric tracks, organized from inner to outer rings: gene locations, with forward strand genes in orange and reverse strand genes in blue; gene density (1/10 k base pair), highlighting regions with high density (>0.7) in purple and low density (<0.3) in orange; repeat density (1/10 k base pair), illustrating areas with high repeat content (>0.7) in blue and low repeat content (<0.3) in red; GC content, marking regions with high GC content (>0.5) in blue and low GC content (<0.3) in red; and GC regions, identifying AT-rich regions (with GC content 0–37.9%) in red and higher GC content (37.9–100%) in cyan. In the center of the plot, the BUSCO completeness assessment of the assembled genome using the Ascomycota lineage data set (ascomycota_odb10) is displayed. Scores indicate the percentages of complete (single-copy and duplicated), fragmented, and missing orthologs.

Our final *L. helva* genome assembly is 30.07 Mb in total length, distributed across 19 contigs, with an N_50_ of 2.68 Mb (Fig. 1). The estimated coverage is 140.99 ×, and the GC content is 46.87%. BUSCO analysis indicated 98.1%. A total of 9,745 protein-coding genes were predicted. Our assembly method, which leverages the strengths of multiple assemblers, will support further work on the adaptation of *L. helva* to mangrove habitats and the conservation of tropical mangrove forest diversity in the face of climate change.

## Data Availability

This Whole Genome Shotgun project has been deposited in DDBJ/ENA/GenBank under the BioProject number PRJNA1209903. The genome accession number for the version described in this announcement is JBKOTY000000000. PacBio reads can be found at the Sequence Read Archive accession number SRR33792350. This study involved sequencing fungal material only and did not require ethical approval by an institutional ethics committee.
